# Prenatal cortisol exposure impairs adrenal function but not glucose metabolism in adult sheep

**DOI:** 10.1530/JOE-23-0326

**Published:** 2024-01-29

**Authors:** K L Davies, J Miles, E J Camm, D J Smith, P Barker, K Taylor, A J Forhead, A L Fowden

**Affiliations:** 1 Department of Physiology, Development and Neuroscience, University of Cambridge, Downing Street, Cambridge, UK; 2 The Ritchie Centre, Hudson Institute of Medical Research, Clayton, Australia; 3 MRC Metabolic Diseases Unit, Mouse Biochemistry Laboratory, Cambridge Biomedical Campus, Cambridge, UK; 4 Endocrine Laboratory, Blood Sciences, Cambridge University Hospitals NHS Foundation Trust, Hills Road, Cambridge, UK; 5 Department of Biological and Medical Sciences, Oxford Brookes University, Oxford, UK

**Keywords:** cortisol, glucose–insulin dynamics, pituitary–adrenal function, developmental programming

## Abstract

Adverse environmental conditions before birth are known to programme adult metabolic and endocrine phenotypes in several species. However, whether increments in fetal cortisol concentrations of the magnitude commonly seen in these conditions can cause developmental programming remains unknown. Thus, this study investigated the outcome of physiological increases in fetal cortisol concentrations on glucose–insulin dynamics and pituitary–adrenal function in adult sheep. Compared with saline treatment, intravenous fetal cortisol infusion for 5 days in late gestation did not affect birthweight but increased lamb body weight at 1–2 weeks after birth. Adult glucose dynamics, insulin sensitivity and insulin secretion were unaffected by prenatal cortisol overexposure, assessed by glucose tolerance tests, hyperinsulinaemic–euglycaemic clamps and acute insulin administration. In contrast, prenatal cortisol infusion induced adrenal hypo-responsiveness in adulthood with significantly reduced cortisol responses to insulin-induced hypoglycaemia and exogenous adrenocorticotropic hormone (ACTH) administration relative to saline treatment. The area of adrenal cortex expressed as a percentage of the total cross-sectional area of the adult adrenal gland was also lower after prenatal cortisol than saline infusion. In adulthood, basal circulating ACTH but not cortisol concentrations were significantly higher in the cortisol than saline-treated group. The results show that cortisol overexposure before birth programmes pituitary–adrenal development with consequences for adult stress responses. Physiological variations in cortisol concentrations before birth may, therefore, have an important role in determining adult phenotypical diversity and adaptability to environmental challenges.

## Introduction

Human epidemiological observations and experimental studies in animals have shown that the intrauterine environment has an important role in determining the adult metabolic and endocrine phenotype (Hales & Barker 2001, Gluckman *et al*. 2008). Suboptimal intrauterine conditions induced experimentally by maternal under- and over-nutrition, hypoxia or placental insufficiency lead to adult metabolic and endocrine dysfunction in a wide range of species (McMillen & Robinson 2005, Reynolds 2013, Hanson & Gluckman 2014). More specifically, there are changes in glucose tolerance, insulin sensitivity and the functioning of the pancreatic β-cells and hypothalamic–pituitary–adrenal (HPA) axis in adulthood following suboptimal conditions *in utero* (Bloomfield *et al.* 2003, Gardner *et al.* 2005, Braun *et al.* 2013, Jellyman *et al.* 2015). Often, but not always, these changes are associated with abnormal birth weight (Moss *et al.* 2001, Gluckman *et al.* 2008, Long *et al.* 2012). Similarly, environmental conditions that alter the intrauterine supply of nutrients and/or oxygen are associated with adult metabolic dysfunction in human populations of diverse ethnicity (Hales & Barker 2001, Gluckman *et al.* 2008). Collectively, these studies have led to the concept that adult metabolic and endocrine function can be programmed developmentally *in utero*.

With many of the prenatal environmental challenges known to programme postnatal phenotype, concentrations of the glucocorticoid stress hormones rise in the maternal and/or the fetal circulations, particularly during late gestation (Reynolds 2013, Hanson & Gluckman 2014). Close to term, glucocorticoids are known to slow fetal growth and induce a variety of structural and functional changes in key fetal tissues essential for neonatal survival (Fowden & Forhead 2015). However, if these glucocorticoid-induced developmental changes are activated earlier in gestation, they could have adverse consequences for metabolic and endocrine function much later in postnatal life (Nathanielsz *et al.* 2003, Jellyman *et al.* 2015). Maternal administration of potent synthetic glucocorticoids during mid-to-late pregnancy has been shown to have long-term metabolic and endocrine consequences for the adult offspring in a range of species, including non-human primates, horses, sheep, guinea pigs, rats and mice (Nyrienda *et al.* 1998, Moss *et al.* 2001, Drake *et al.* 2005, de Vries *et al.* 2007, Vaughan *et al.* 2015, Valenzuela *et al.* 2017, McGowan & Matthews 2018). Long-term follow-up studies of human infants whose mothers received synthetic glucocorticoids during pregnancy also indicate a higher incidence of metabolic maladaptation as this population ages (Entringer *et al.* 2009, Bosch *et al.* 2012, Martin *et al.* 2021). In pregnant sheep, maternal stress and administration of synthetic glucocorticoids have been shown to lead to glucose intolerance, insulin insensitivity and altered function of the pancreatic β-cells and HPA axis in the adult offspring (Moss *et al.* 2001, Sloboda *et al.* 2002, Long *et al.* 2012, Wei *et al.* 2023). In some instances, these changes persist into the next generation (Drake *et al.* 2005, Long *et al.* 2012) However, relatively little is known about the long-term effects of naturally occurring increments in fetal cortisol concentrations of the magnitude seen in response to prenatal environmental challenges known to programme adult metabolic and endocrine phenotypes. Thus, this study examined the hypothesis that raising fetal cortisol concentrations within the physiological range in fetal sheep before term would impair their glucose–insulin dynamics and HPA axis function in adulthood.

## Methods

### Animals

All animal procedures were carried out under the UK Animals (Scientific Procedures) Act 1986 Amendment Regulations 2012, following ethical approval by the Animal Welfare and Ethical Review Body of the University of Cambridge. A total of 18 time-mated pregnant Welsh Mountain ewes with single fetuses were utilised in this study. The pregnant ewes were group-housed in barns before surgery and single-housed within the sight and sound of other sheep after surgery until spontaneous labour and delivery. The ewes and their newborn lambs were barn-housed for a further 4–6 weeks before moving to grazing. The 18 offspring studied as adults were weaned at 12 weeks of postnatal age and subsequently kept for grazing with vitamin and mineral supplements available *ad libitum*. A week before surgery as young adults, they were returned to single housing within sight and sound of other sheep until the end of the experimental protocol. When housed indoors, the pregnant ewes and their adult offspring had free access to hay and water, except for 12–18 h before surgery when food was withheld.

### Surgical procedures

Between 114 and 119 days of gestational age (dGA, term approximately 145 dGA), surgery was carried out on the ewes under isoflurane anaesthesia (1.5–2% in 5:1 O_2_:N_2_O mixture) with positive pressure ventilation. Catheters were inserted into the maternal dorsal aorta and the fetal caudal vena cava via the maternal femoral artery and two branches of the fetal tarsal vein, respectively, and then exteriorised through the maternal flank. In the adult offspring, a catheter was inserted into the dorsal aorta, and two catheters were placed into the caudal vena cava via the femoral vessels using the same anaesthetic procedure as for the pregnant ewes. All animals were monitored throughout surgery using a capnograph and pulse oximeter. At surgery, they were given antibiotics (oxytetracycline, 20 mg/kg i.m., Alamycin, Norbrook Laboratories, Newry, UK; penicillin, Depocillin, Intervet International, Milton Keynes, UK, 15 mg/kg i.m. to adults and Crystapen 200 mg i.v. to fetus) and analgesia (carprofen, 1 mg/kg s.c. to the adults, Rimadyl, Zoetis, London, UK). Adult penicillin treatment continued for 2 days post operation.

### Experimental procedures

#### Fetal cortisol treatment

After catheterisation, all animals were sampled daily to maintain catheter patency and to collect blood samples to measure blood gases, metabolite concentrations and plasma hormone concentrations. Following maternal post-operative recovery for at least 5 days, the catheterised fetuses were assigned randomly to receive a 5-day intravenous infusion of either saline (0.9% NaCl, 3 mL/day, *n* = 9, controls, 4 males [M]: 5 females [F]) or cortisol (1–2 mg/kg/day Solu-Cortef, Pharmacia, *n* = 9, 4M:5F) with respect to balancing the numbers of males and females in each treatment group. The dose of cortisol was chosen to cause a three- to four-fold increase in fetal cortisol concentrations (Table 1), in line with the cortisol increments seen previously in sheep fetuses in response to suboptimal intrauterine conditions induced by maternal undernutrition, hypoxia, placental insufficiency and cord occlusion in late gestation (Nathanielsz *et al.* 2003, Fowden & Forhead 2015).

**Table 1:** Mean (± s.e.m.) values of plasma cortisol concentration in the fetuses before (pre-infusion) and during the 5 days of infusion and in the adults at the end of the experimental studies, together with body weights at birth, during sucking to adulthood and fractional growth rates into adulthood in sheep infused with cortisol or saline *in utero* (*n* = 9 in both treatment groups).

	Saline	Cortisol
Cortisol(nmol/L)		
Fetus		
Pre-infusion	29 ± 3	33 ± 4
During infusion	33 ± 4	114 ± 9^c,d^
Adult	29 ± 5	32 ± 7
Body weight (kg)		
Birth	3.37 ± 0.17	3.68 ± 0.12
1 week	4.79 ± 0.27	5.64 ± 0.25^b^
2 weeks	6.11 ± 0.30	7.10 ± 0.29^b^
1 month	9.48 ± 0.70	11.01 ± 0.62
3 months	21.1 ± 0.8	23.8 ± 1.2
Adult	37.2 ± 1.6	38.7 ± 1.7
Fractional growth rate (kg/week/kg starting wt)		
Birth–1 week	0.42 ± 0.02	0.54 ± 0.06^a^
1–2 weeks	0.28 ± 0.05	0.30 ± 0.05
Birth–1 month	0.45 ± 0.03	0.51 ± 0.06
Birth–3 months	0.44 ± 0.02	0.47 ± 0.04
Birth–adulthood	0.28 ± 0.01	0.28 ± 0.02

Significant difference between saline and cortisol treatment groups (letters a, b, c) and significant increment during infusion (letter d) shown.

^a^*P* = 0.09 (*t*-test); ^b^*P* < 0.05 (*t*-test); ^c^*P* < 0.01 (*t*-test); ^d^*P* < 0.01 (paired *t*-test).

At the end of infusion (128–131 dGA), the maternal catheter was removed by gentle traction in the conscious state, and the fetal catheters were shortened, sealed and internalised naturally after disinfection. The ewes were allowed to deliver spontaneously and killed after weaning their lambs by the administration of a lethal dose of anaesthetic (200 mg/kg sodium pentobarbitone i.v., Pentoject, Animalcare Ltd., York, UK).

#### Measurements in juvenile offspring

At birth, the lambs were weighed. Any catheters remaining *in situ* in the lambs were removed by gentle traction under local anaesthesia, and all but one of the cortisol-infused ram lambs were castrated by ringing the scrotum shortly after birth. The remaining ram lamb had undescended testes at birth and was surgically castrated under anaesthesia at 12 weeks of age by the Named Veterinary Surgeon. All lambs were weighed weekly from birth to 4 weeks and then monthly to 8 months. Fractional growth rate was calculated as the increment in body weight over a set period of time divided by the body weight at the beginning of the period. The mean postnatal age at re-catheterisation as adults was similar in the two treatment groups (saline, 43.8 ± 1.88 ± 1.8 weeks; cortisol, 44.9 ± 1.3 weeks, both *n* = 9).

#### Adult metabolic and endocrine challenges

After at least 2–3 days of post-operative recovery, a series of four metabolic and endocrine challenge tests were carried out in the adults at intervals of 2–4 days: an intravenous glucose tolerance test, a hyperinsulinaemic–euglycaemic challenge test and an insulin-induced hypoglycaemic challenge test in a random order followed by an adrenocorticotropic hormone (ACTH) challenge test. Blood samples for measurement of hormone concentrations were collected into heparin- and/or EDTA-coated tubes, and after centrifugation, the plasma was stored at −20 °C for subsequent analyses.

##### Intravenous glucose tolerance test

After fasting overnight, glucose was infused over 5 min into the venous catheter (0.5 g/kg, 50% dextrose solution, Arnold, Shrewsbury, UK). Arterial blood samples were taken at 5–10-min intervals from 10 min before to 100 min after starting the infusion and then again at 120 min. Glucose tolerance was assessed as the area under curve of the glucose increment (AUCG), while insulin secretion was measured as the area under curve of the insulin increment (AUCI) above the respective basal, pre-infusion values. Relative insulin secretion was calculated as AUCI divided by AUCG.

##### Hyperinsulinaemic–euglycaemic clamp

After fasting overnight, a bolus of insulin (approximately 10–12 pmol in 1 mL saline, Actrapid human insulin, Novo Nordisk) was given intravenously via one of the venous catheters followed immediately by a continuous infusion for 2 h (48 pmol insulin/kg/min). After 15 min of insulin infusion, glucose (25% dextrose solution, Arnold, Shrewsbury, UK) was infused via the other venous catheter at a known variable rate to maintain blood glucose levels at the mean glucose concentration (±5%) measured over the 30-min basal period before insulin administration. Arterial blood samples (0.2 mL) were taken for blood glucose measurements every 5 min with larger samples (5 mL) drawn for the measurement of plasma insulin concentrations immediately before infusion and again at 90, 105 and 120 min after starting the insulin infusion once steady state had been achieved. Insulin sensitivity of glucose metabolism was measured as the steady-state rate of glucose infusion (μmol/kg/min) during the second hour of insulin infusion divided by the steady-state insulin concentration during this period (pmol/L). Insulin clearance was calculated as the rate of insulin infusion (48 pmol/kg/min) divided by the steady-state insulin concentration (pmol/L).

##### Insulin-induced hypoglycaemic challenge test

Hypoglycaemia was induced in the fed state by intravenous administration of a bolus dose of insulin (5.25 μg/kg in 10 mL saline, Actrapid human Insulin, Novo Nordisk). Arterial blood samples (1 mL) were taken at 5–10-min intervals for 30 min before to 60 min after insulin administration to monitor blood glucose concentrations, with larger samples (5 mL) taken immediately before and at 60 min after the insulin bolus in a subset of each treatment group to measure the plasma cortisol and ACTH concentrations.

##### ACTH challenge test

An intravenous bolus of ACTH (1.25 μg/kg, synacthen, Alliance Pharmaceuticals Ltd., Wiltshire, UK) was given in the fed state, and arterial blood samples (3–4 mL) were taken immediately before and then at 30, 45, 60, 90 and 120 min after ACTH administration to measure plasma cortisol concentrations.

#### Tissue collection

At the end of the experimental period, tissues were collected in the fed state after euthanasia using a lethal dose of anaesthetic, as described previously. A range of tissues were collected to provide fresh and frozen tissue for this and other studies (Davies *et al.* 2023). For this study, the adrenal glands of all animals were weighed, and the right gland from a subset of each treatment group was fixed in 4% paraformaldehyde (with 0.2% glutaraldehyde in 0.1 M phosphate buffer, pH 7.3) for histological analyses.

### Biochemical analyses

Fetal cortisol concentrations were measured using a human ELISA (RE52061, Tecan, Männedorf, Switzerland), previously validated for sheep plasma (Vaughan *et al.* 2016). Intra- and inter-assay coefficients of variation for the cortisol assay were 3% and 8%, respectively, and the limit of detection was 4 pmol/L. Cortisol concentrations in adult plasma were analysed by liquid chromatography–mass spectrometry using a Sciex 5500 Triple Quad mass spectrometer in positive ionisation mode. Chromatography was performed using a Shimadzu chromatography system in conjunction with a phenyl hexyl stationary phase column. Inter-assay coefficients of variation were 3.7, 5.3 and 4.4% at concentrations of 93, 433 and 725 nmol/L, respectively. The lower limit of detection was 5 nmol/L. Ovine insulin was measured using an ELISA assay (Mercodia Ovine Insulin Elisa, Mercodia, Uppsala, Sweden). Intra- and inter-assay coefficients of variation for the ovine insulin assay were 3% and 9%, respectively, and the limit of detection was 5 pmol/L. Plasma concentrations of human insulin during the HEC challenge were measured using a human chemiluminescence immunoassay (DiaSorin, Saluggia, Italy) by the MRC MDU Mouse Biochemistry Laboratory (MC_UU_00014/5). The inter-assay coefficients of variation were 11.0% at 34 pmol/L, 7.0% at 135 pmol/L, 6.7% at 365 pmol/L and 5.9% at 1024 pmol/L, and the minimum detectable level was 3 pmol/L. Plasma ACTH concentrations were measured in a single assay using an ELISA kit (ACTH1-39; Demeditec Diagnostics GmbH, Kiel, Germany), as described previously (Camm *et al.* 2021). The intra-assay coefficient of variation was 6%, and the minimum detection level was 0.22 pg/mL. Blood and glucose concentrations were measured using an automated analyser (Yellow Springs 2300 Stat Plus glucose/lactate analyser, YSI Ltd, Farnborough, UK).

### Histological analyses

After paraformaldehyde fixation of the adrenal gland for 2 days, it was transferred into phosphate-buffered saline and stored at 4 °C until analysis. The adrenal was cut transversely approximately at the midline and one-half embedded in paraffin wax. Five groups of ten 7 μm sections were cut transversely at the midline of the adrenal gland at intervals of 150 μm, and the sections floated in a water bath before loading onto electrostatically charged microscope slides. Slides were stained with haematoxylin and eosin to distinguish the cortical and medullary zones, and imaged and analysed using a Nanzoomer scanner (Nanozommer 2.0-RS 010739 Series, Hamamatsu Photonics, UK, and NDP view software). The total transverse area of the whole adrenal and the areas of the capsule, cortex, medulla and the interdigitation between cortical and medullary cells were measured in each section blindly to treatment groups and averaged per adrenal gland.

### Statistical analysis

Data are presented as mean ± s.e.m. with Sigma Stat 3.5 used for statistical analyses (Systat Software Inc, Point Richmond, CA, USA). Differences between cortisol- and saline-treated animals were analysed by Student’s *t*-test or non-parametric Mann–Whitney test, as appropriate, with the males and females in each treatment group combined due to the small sample size for sex differences and male castration. *P* < 0.05 was considered significant throughout.

## Results

### Prenatal cortisol concentrations and postnatal morphometric measurements

The average concentration of plasma cortisol during infusion was significantly higher in the cortisol-treated group than saline-treated fetuses, with no significant difference in basal concentrations between the two treatment groups before infusion (Table 1). There was no effect of prenatal treatment on birth weight (Table 1). However, at 1 and 2 weeks of postnatal age, lambs treated prenatally with cortisol were significantly heavier, with a trend for a higher fractional growth rate over the first postnatal week, than in their saline-infused counterparts (Table 1). There were no further differences in body weight or fractional growth rate between the two treatment groups with advancing age (Table 1).

### Glucose–insulin dynamics

#### Glucose tolerance

**Figure 1:**
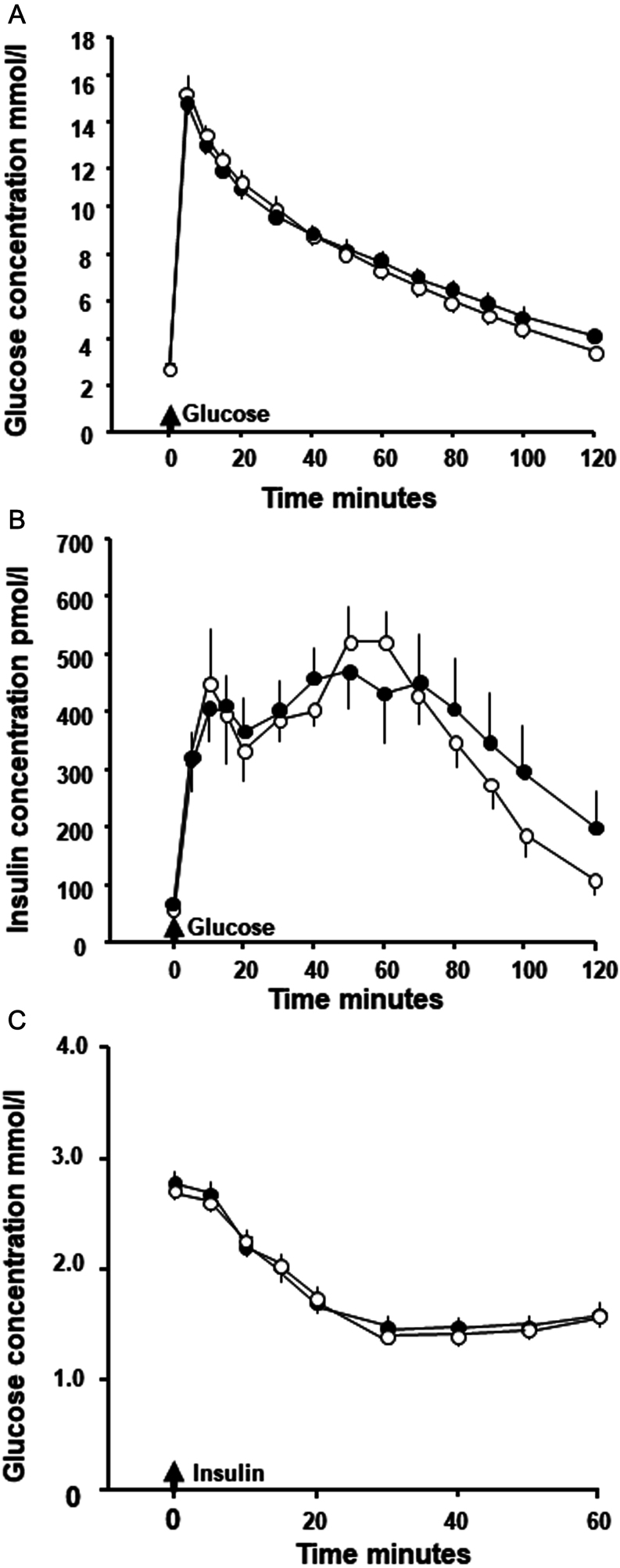
Mean (± s.e.m.) concentrations of (A) blood glucose and (B) plasma insulin during a glucose tolerance test (GTT) and of (C) blood glucose in the insulin tolerance test (ITT) in adult sheep that had been infused prenatally with either saline (open symbols) or cortisol (filled symbols) between 125 and 130 days of gestation (GTT: Saline *n* = 8 and Cortisol *n* = 9; ITT: *n* = 9 in both groups).

Adult glucose tolerance was unaffected by prenatal treatment (Fig. 1A). The basal and peak glucose concentrations as well as the AUCG were not significantly different between prenatal treatments (Table 2). Similarly, adult insulin secretion was unaffected by prenatal treatment (Fig. 1B). There were also no significant differences in basal or peak insulin concentrations, the AUCI or in the relative insulin secretion between prenatal treatments (Table 2). However, insulin concentrations remained significantly elevated above the basal value at 120 min after glucose administration in adults infused prenatally with cortisol but not saline (Increment: Saline, +47 ± 33 pmol/L, *n* = 8, *t* = 2.13, *P* > 0.05; Cortisol, +134 ± 54 pmol/L, *t* = 2.39, *n* = 9, *P* < 0.05; *t*-test, significance of a single mean differing from zero). The half-time for glucose clearance also tended to be longer in the cortisol-treated group than saline-treated group, but this did not reach statistical significance (Table 2).

**Table 2:** Mean (± s.e.m.) arterial concentrations of blood glucose and plasma insulin and derived measures of glucose–insulin dynamics during the glucose tolerance test, the hyperinsulinaemic–euglycaemic clamp and the insulin tolerance test in adult sheep treated with either saline or cortisol *in utero*.

	Saline	Cortisol
**Glucose tolerance test**		
	*n* = 8	*n* = 9
Basal glucose (mmol/L)	2.86 ± 0.12	2.85 ± 0.10
Basal insulin (pmol/L)	60 ± 9	69 ± 10
Peak glucose (mmol/L)	15.6 ± 0.6	14.9 ± 0.5
Peak insulin (pmol/L)	594 ± 75	578 ± 80
AUC glucose (mmol/L/min)	588 ± 45	608 ± 44
AUC insulin (pmol/L/min)	34198 ± 3285	36367 ± 6217
Relative insulin secretion (pmol/mmol)	60.4 ± 7.3	60.1 ± 8.0
*t*½ glucose clearance (min)	36.1 ± 2.6	44.2 ± 3.0^a^
**Hyperinsulinaemic–euglycaemic clamp**		
	*n* = 9	*n* = 9
Basal glucose (mmol/L)	2.84 ± 0.10	2.96 ± 0.13
Steady state glucose (mmol/L)	2.81 ± 0.10	2.93 ± 0.12
Steady state insulin (pmol/L)	14075 ± 959	13626 ± 782
Steady state glucose infusion rate (μmol/min/kg)	19.1 ± 1.8	17.6 ± 0.8
Insulin sensitivity of glucose metabolism (μmol/L/nmol/kg/min)	1.46 ± 0.20	1.35 ± 0.14
Insulin clearance (mL/min/kg)	3.60 ± 0.30	3.60 ± 0.20
**Insulin tolerance test/insulin-induced hypoglycaemia**		
	*n* = 9	*n* = 9
Basal glucose (mmol/L)	2.75 ± 0.08	2.80 ± 0.09
Nadir glucose (mmol/L)	1.30 ± 0.08	1.40 ± 0.09
AAC glucose (mmol/L/min)	58.1 ± 4.0	59.4 ± 1.5
	*n* = 8	*n* = 7
Basal ACTH (pg/mL)	5.3 ± 0.5	6.5 ± 0.5^b^
ACTH increment (0–60 min pg/mL)	10.3 ± 3.1	13.7 ± 4.1
Basal cortisol (nmol/L)	23 ± 5	29 ± 7
Cortsiol increment (0–60 min nmol/L)	152 ± 18	76 ± 21^b^

Significant difference between the saline- and cortisol-treated groups shown by letters a and b.

^a^*P* < 0.068; ^b^*P* < 0.02 (*t*-test).

*n*, number of animals.

#### Insulin sensitivity and clearance

In the HEC, basal- and steady-state glucose concentrations did not differ significantly within or between treatment groups (Table 2). Steady-state insulin concentrations were also similar in the treatment groups (Table 2). The adult weight-specific rate of glucose infusion at steady state, insulin sensitivity of glucose metabolism and the rate of insulin clearance did not differ between prenatal treatments (Table 2). Acute insulin administration produced a similar profile and degree of hypoglycaemia in the two groups (Fig. 1C). There were no significant effects of prenatal treatment on the basal or nadir glucose concentrations, nor on the area above the glucose curve (AAC) in response to insulin administration (Table 2).

### Pituitary–adrenal axis function

#### Response to hypoglycaemia

**Figure 2:**
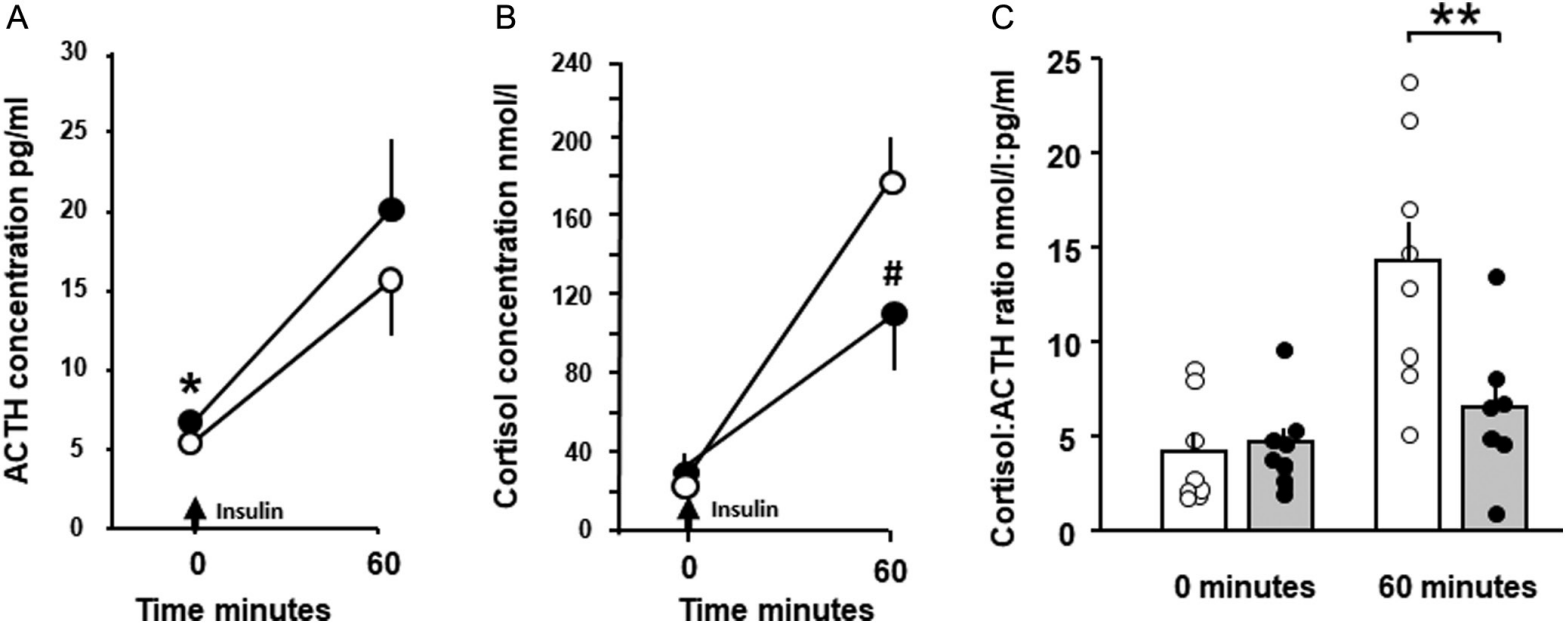
Mean (± s.e.m.) plasma concentrations of (A) ACTH and (B) cortisol and (C) the mean (± s.e.m.) and individual cortisol:ACTH concentration ratios before (0 min) and 60 min after the onset of insulin-induced hypoglycaemia in adult sheep that had been infused prenatally with either saline (open symbols and columns) or cortisol (filled symbols and columns) between 125 and 130 days of gestation (Saline *n* = 8; Cortisol *n* = 7). Significantly different from values in saline-treated group ** *P* < 0.02, *P < 0.05, ^#^ *P* = 0.067 (*t*-test).

Insulin-induced hypoglycaemia increased plasma concentrations of ACTH and cortisol in both treatment groups (Fig. 2A and B, Table 2). The increment in ACTH concentration between the 0-min and 60-min samples did not differ significantly with prenatal treatment (Table 2), although basal ACTH concentrations were significantly higher in the adults that received cortisol prenatally (Fig. 2A, Table 2). Cortisol concentrations were similar in the two treatment groups before insulin administration (Table 2, Fig. 2B), but tended to be lower in cortisol- than the saline-treated animals 60 min after administration (*P* = 0.067, Fig. 2B), despite a similar degree of hypoglycaemia (Table 2, Fig. 1C). However, the cortisol increment in response to hypoglycaemia was significantly less in adults treated prenatally with cortisol than saline (Table 2). When the cortisol to ACTH concentration ratios were calculated, there was a significantly lower concentration ratio in the cortisol- than the saline-treated group in the hypoglycaemic state at 60 min after but not before insulin administration (Fig. 2C).

#### Response to ACTH

**Figure 3:**
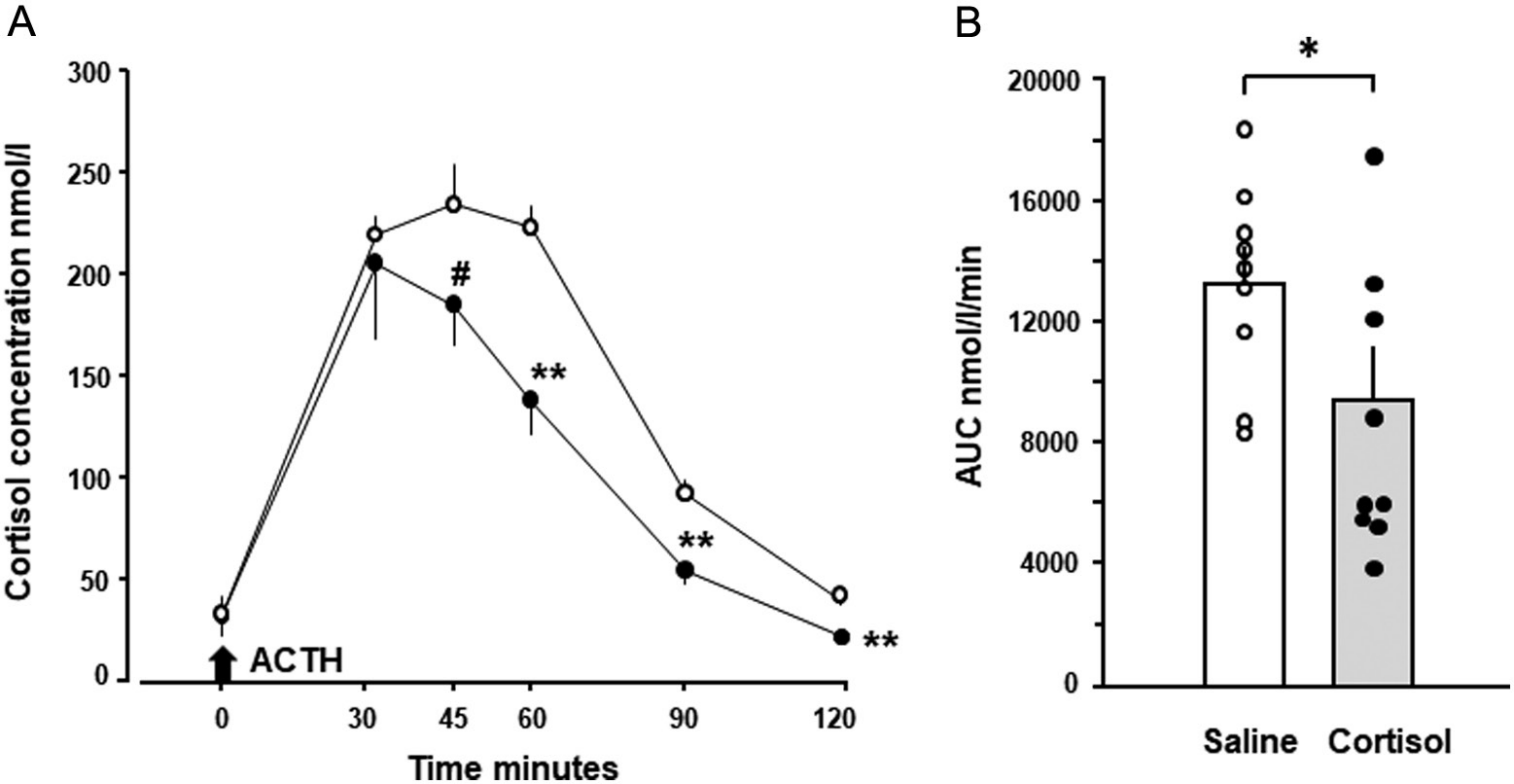
Mean (± s.e.m.) values of (A) plasma concentrations of cortisol and (B) individual and mean (s.e.m.) area under the cortisol curve in response to ACTH administration in adult sheep that had been infused prenatally with either saline (*n* = 9, open symbols and columns) or cortisol (*n* = 9, filled symbols and columns) between 125 and 130 days of gestation. Significantly different from values in the saline-treated group ^#^ *P* = 0.098, *P < 0.05, **P < 0.01 (*t*-test).

Basal cortisol concentrations before ACTH administration were unaffected by prenatal treatment (Saline, 33.4 ± 6.14 ± 6.1 nmol/L; Cortisol, 32.7 ± 9.5 nmol/L, both *n* = 9). The initial cortisol response to ACTH was similar in the two groups, but concentrations declined more rapidly in adults prenatally treated with cortisol than saline (Fig. 3A). Cortisol concentrations were significantly lower in the cortisol- than the saline-treated group from 60 to 120 min after ACTH administration, with a similar trend at 45 min (Fig. 3A). Consequently, the area under the cortisol curve in response to ACTH was significantly smaller in adults prenatally treated with cortisol than saline (Fig. 3B).

#### Adrenal morphology

**Figure 4:**
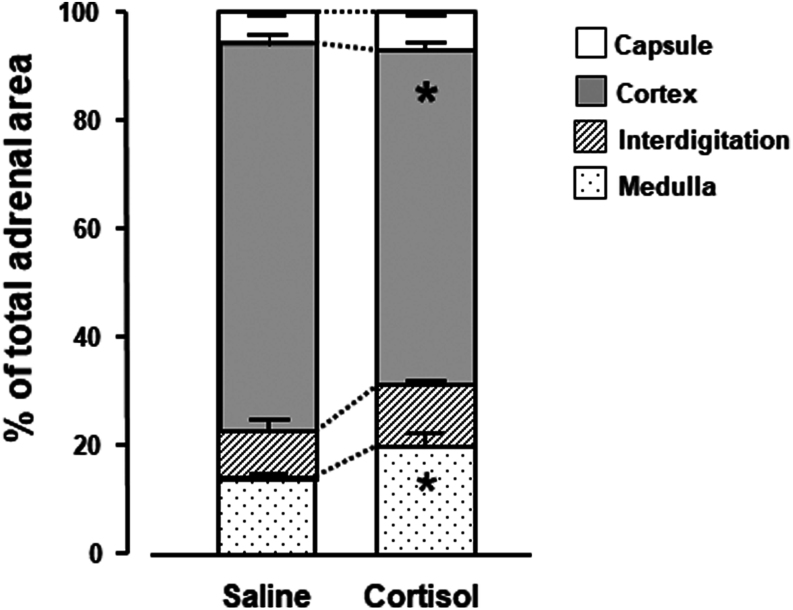
Mean (± s.e.m.) areas of the zones within the adrenal gland expressed as a percentage of the total cross-sectional area of the gland at the mid-transverse plane in adult sheep infused prenatally with either saline (*n* = 6) or cortisol (*n* = 5) between 125 and 130 days of gestation. Significantly different from the values in the saline-treated group *P < 0.05 (*t*-test).

Prenatal treatment had no significant effect on adrenal gland weight in adulthood nor on its total cross-sectional area at the transverse midline plane (Table 3). Compared with saline treatment, the area of the adult adrenal gland that was purely medulla was significantly greater with prenatal cortisol treatment (Table 3). None of the other adrenal zones differed in absolute area with prenatal treatment (Table 3). However, when the individual zone areas were expressed as a percentage of the total cross-sectional area, the area that was pure cortex was significantly smaller, while the area of pure medulla was significantly greater in adults prenatally treated with cortisol than saline (Fig. 4).

**Table 3:** Mean (± s.e.m.) weight of both adrenal glands, total area and the areas of the different zones of the right adrenal at the transverse midline plane in adults treated with saline or cortisol *in utero*.

	Saline	Cortisol
	*n* = 9	*n* = 9
Total weight (g)	2.22 ± 0.26	2.22 ± 0.16
**Area (mm^2^)**	*n* = 6	*n* = 5
Total area	35.9 ± 3.2	36.7 ± 2.7
Capsule	2.2 ± 0.2	2.5 ± 0.2
Cortex	26.1 ± 2.1	22.9 ± 1.8
Cortical–medullary inter-digitation	3.2 ± 1.0	4.2 ± 0.5
Medulla	4.5 ± 0.4	7.3 ± 1.0^a^

^a^Significantly different from the value in the saline-treated groups (*t*-test, *P* < 0.05).

*n*, number of individuals.

## Discussion

The results show that a physiological increase in cortisol concentrations in fetal sheep for a short period in late gestation impairs pituitary–adrenal function, but has little apparent effect on glucose–insulin dynamics in adulthood. The adult adrenocortical response to hypoglycaemia was reduced after prenatal cortisol treatment in association with reductions in adrenal ACTH sensitivity and the relative area of the adrenal cortex. These changes in the adrenal glands were accompanied by minor changes in body growth during the immediate neonatal period, although birth weight was unaffected by prenatal treatment. These results show that exposure to excess cortisol during the sensitive period of prepartum tissue maturation can have consequences for stress responsiveness much later in postnatal life. These findings have implications for the developmental programming of the HPA axis by other environmental challenges of fetal or maternal origin that raise fetal cortisol concentration naturally during late gestation.

Maternal treatment with synthetic or natural glucocorticoids during late pregnancy has been shown previously to impair glucose–insulin dynamics in postnatal offspring of several species (Nyrienda *et al.* 1998, Moss *et al.* 2001, Kanitz *et al.* 2006, de Vries *et al.* 2007, Sloboda *et al.* 2007, Long *et al.* 2012, Reynolds 2013, Vaughan *et al.* 2015, Valenzuela *et al.* 2017). In pregnant ewes, administration of potent synthetic glucocorticoids in the last-third pregnancy is known to cause glucose intolerance, insulin insensitivity and/or decreased insulin secretion with increasing age of their offspring (Moss *et al.* 2001, Sloboda *et al.* 2002, 2005, 2007, Long *et al.* 2012). In contrast, direct intramuscular treatment of fetal sheep with synthetic glucocorticoids in late gestation has little or no effect on adult glucose–insulin dynamics compared with maternal treatment even at 3.5 years of age (Moss *et al.* 2001, Sloboda *et al.* 2005). In this study, a physiological increment in the cortisol concentration for 5 days in fetal sheep in late gestation also had no apparent effect on glucose–insulin dynamics in young adulthood. Adult glucose tolerance, relative insulin secretion and insulin clearance measured in the animals catheterised *in utero* were also similar to values observed previously in young adult sheep that received no prenatal interventions (Cowett *et al.* 1980, Gardner *et al.* 2005). Collectively, the studies in sheep suggest that the route of fetal glucocorticoid exposure is an important determinant of glucose-insulin dynamics in adulthood. This may relate, in part, to differences in intrauterine growth as birth weight is reduced with maternal but not direct fetal treatment in late gestation in this and previous studies (Moss *et al*. 2001, Nathanielsz *et al.* 2003, Jensen *et al.* 2005, Vaughan *et al.* 2018).

Compared with rodent and primate species (Drake *et al.* 2005, de Vries *et al.* 2007, Reynolds 2013, McGowan & Matthews 2018), sheep appear to be less sensitive to prenatal glucocorticoid programming of their adult insulin–glucose dynamics, probably because their adult metabolism depends more heavily on volatile fatty acids than glucose (Judson *et al.* 1976). Indeed, in the current cohort of adult sheep, a previous study has shown alterations in mitochondrial substrate utilisation of fatty acids, but not glucose, in specific skeletal muscles prenatally treated with cortisol (Davies *et al.* 2023). Thus, preterm increases in fetal cortisol concentrations within the physiological range may have adult metabolic consequences in sheep, but these may relate principally to metabolites other than glucose.

Exogenous administration of natural and potent synthetic glucocorticoids to mothers in late pregnancy also alters HPA function of their adult offspring in several species, including sheep (Sloboda *et al.* 2005, 2007, Braun *et al.* 2013, Jellyman *et al.* 2015, Vaughan *et al.* 2016). Similarly, exposure to environmental stressors that raise maternal glucocorticoid concentrations endogenously also leads to HPA dysfunction in their offspring postnatally (Bloomfield *et al.* 2003, Gardner *et al.* 2005, McGowan & Mathews 2018). Both hypo- and hyper-reactivity of the postnatal HPA axis have been observed in these studies, depending on the species, age of the offspring, and on the type, dose, duration and timing of the maternal glucocorticoid overexposure (Li *et alet al.* 2013, Howland *et al.* 2017, McGowan & Matthews 2018, Martin *et al.* 2021). In human populations, the response of the adult HPA axis to prenatal glucocorticoid overexposure appears to switch from hyper-responsiveness in pre-pubescent children to hypo-responsiveness in young adults with reduced responses to both stressful stimuli and exogenous ACTH administration (Entringer *et al.* 2009, Bosch *et al.* 2012, Howland *et al.* 2017, Weiss *et al.* 2023). A similar decline in postnatal HPA responsiveness with increasing age has also been observed in guinea pigs and sheep after prenatal exposure to synthetic glucocorticoids via maternal treatment (Liu *et al.* 2001, Sloboda *et al.* 2002, 2007). Collectively, human epidemiological and experimental animal studies have shown that overexposure to maternal glucocorticoids before birth can programme postnatal HPA dysfunction via effects on the sensitivity of all levels of the HPA axis, with consequences for the set point, forward drive and negative feedback regulation of the axis (Li *et al.* 2013, McGowan & Matthews 2018, Martin *et al.* 2021).

In this study, raising cortisol levels within the physiological range specifically in the fetus led to a reduced cortisol response to both hypoglycaemia and ACTH administration in adulthood. This adrenal hypo-responsiveness was associated with a percentage reduction in the cortical area of the adult adrenal gland and occurred without any significant difference in the incremental or absolute ACTH concentrations during hypoglycaemia. Although adult adrenal weight was unaffected by fetal cortisol treatment in this study, a previous study has shown an altered trajectory of adrenal growth *in utero* in response to cortisol infusion with impaired growth during infusion followed by rebound growth to achieve a greater-than-normal adrenal weight 5 days post infusion (Vaughan *et al*. 2018). Abundance of the ACTH receptor in the fetal ovine adrenal is decreased by fetal cortisol infusion during late gestation (Wang *et al.* 2004), although whether this persists into adulthood remains unknown. No changes in ACTH receptor abundance are observed in adrenal glands of adult sheep after direct fetal betamethasone treatment (Sloboda *et al.* 2007). In addition, neither maternal nor fetal treatment with synthetic glucocorticoids in late gestation appears to influence gene expression for key adrenal steroidogenic enzymes in the offspring (Sloboda *et al.* 2007, Li *et al.* 2013). However, further studies are needed to determine whether prenatal cortisol overexposure affects ovine adrenal steroidogenic pathways in adulthood.

In fetal sheep, cortisol infusion reduces adrenal abundance of insulin-like growth factor (IGF)-II, a major fetal growth factor that is expressed predominantly in the zona fasciculata of fetal ovine adrenal glands near term (Han *et al*. 1992, Lü *et al.* 1994, Coulter *et al.* 2002). In turn, this may impair cortical differentiation of the juxtacortical cells in the interdigation zone of the fetal ovine adrenal normally seen in late gestation (Boshier *et al.* 1989). This may allow expansion of the adrenal medulla, in line with the increased medullary area observed in the adult adrenal gland after prenatal cortisol treatment in this study. Growth of the adrenal medullary region may also have been stimulated by upregulated abundance of IGF-I, a key postnatal growth factor known to be expressed in the fetal adrenal medulla and upregulated in other fetal tissues by cortisol infusion (Li *et al.* 1996, Camm *et al.* 2020). Adrenal medullary expansion at the expense of the cortex in response to fetal cortisol administration is also consistent with the reduced zona fasciculata volume found in juvenile mice after maternal corticosterone treatment and with the increase in noradrenaline concentration, adrenal phenylethanolamine N-methyl transferase abundance and in sympatho-adrenal activation seen postnatally in several species following prenatal glucocorticoid overexposure (Kanitz *et al.* 2006, Shaltout *et al.* 2011, Cuffe *et al*. 2017, Khurana *et al.* 2019).

Cortisol is known to have a negative feedback effect on the fetal ovine HPA axis and reduces corticotrophin-releasing hormone (CRH) receptor expression in the fetal ovine pituitary in late gestation (Green *et al.* 2000, Holloway *et al.* 2001, Wood & Walker 2015). In contrast, there are few changes in the basal abundance of hypothalamic CRH, pituitary proopiomelanocortin (POMC), POMC cleavage enzymes, glucocorticoid receptor or in the type of circulating ACTH in fetal sheep in response to cortisol infusion in late gestation (Ozolins *et al.* 1991, Matthews & Challis 1995, Matthews *et al.* 1995, Holloway *et al.* 2001). Collectively, these observations suggest that the reduced adrenocortical responsiveness of the adult sheep prenatally treated with cortisol in this study is more likely to be due to changes in the adrenal gland than in the central drive of the HPA axis. However, basal ACTH concentrations were higher in adult sheep receiving cortisol prenatally without any significant difference in their basal cortisol concentrations. Consequently, there may have been some degree of central resetting of the adult HPA axis by fetal cortisol overexposure, in addition to the adrenal changes in these adults. With direct administration of synthetic glucocorticoids to fetal sheep, there is more evidence for pituitary as well as adrenal involvement in the programming of the adult HPA, although these effects vary with increasing postnatal age (Sloboda *et al.* 2002, 2007, Li *et al*. 2013). Further studies are, therefore, needed to determine whether the central regulation of HPA activity is programmed in adult sheep by physiological variations in the fetal cortisol concentration.

The mechanisms by which prenatal cortisol overexposure programmes adult phenotype may be direct or mediated indirectly by other factors that regulate fetal development. Overexposure to synthetic and natural glucocorticoids in late pregnancy is known to alter both maternal dietary intake and the placental capacity for nutrient transfer with consequences for the fetal nutrient supply (Jensen *et al.* 2005, Gnanalingham *et al.* 2008, Vaughan *et al.* 2016, 2018). Furthermore, cortisol influences the fetal availability of several other growth-regulatory hormones and growth factors, in addition to the IGFs (Fowden & Forhead 2015). For instance, in sheep, maternal dexamethasone treatment and fetal cortisol infusion both affect fetal concentrations of the thyroid hormones and leptin (Forhead *et al.* 2007, Fowden & Forhead 2015). In turn, these hormones have actions on metabolism, tissue differentiation and the development of other endocrine systems, including the fetal HPA axis (O’Connor *et al.* 2007, Harris *et al.* 2017, Davies *et al.* 2020, 2021, Camm *et al.* 2021). In addition, glucocorticoids may influence sex hormone concentrations *in utero* with potential effects on endocrine and metabolic development (Cardoso & Padmanabhan 2019, Sheng *et al.* 2020). In adulthood, testosterone is known to suppress HPA function and alter glucose–insulin dynamics in several species, but postnatal variations in the testosterone concentration are unlikely to account for the current findings as all the males were castrated (Rubinow *et al.* 2005, Sheng *et al.* 2020). Developmental programming by prenatal glucocorticoid overexposure is, therefore, likely to be multifactorial in origin, with effects on multiple physiological systems, including several endocrine axes.

In conclusion, physiological increases in fetal cortisol concentrations commonly seen in response to short-term environmental challenges during late gestation have little apparent effect on adult glucose–insulin dynamics but can programme development of the HPA axis, with consequences for stress responsiveness much later in adult life. Thus, naturally occurring variations in prenatal cortisol exposure are likely to contribute to the phenotypical diversity of adult populations and their ability to adapt to environmental challenges.

## Declaration of interest

The authors declare that there are no conflict of interest that could be perceived as prejudicing the impartiality of the research reported.

## Funding

This study was funded by a Biotechnology and Biological Sciences Research Councilhttp://dx.doi.org/10.13039/501100000268 grant (BB/P019048/1). The data are stored in the University of Cambridgehttp://dx.doi.org/10.13039/501100000735 repository with the reference access https://doi.org/10.17863/CAM.104324 after publication.
